# Usefulness of Daily Fractionated Administration of Wortmannin Combined With γ-Ray Irradiation in Terms of Local Tumor Response and Lung Metastasis

**DOI:** 10.4021/wjon640w

**Published:** 2013-03-06

**Authors:** Shin-ichiro Masunaga, Yoshinori Sakurai, Hiroki Tanaka, Minoru Suzuki, Natsuko Kondo, Masaru Narabayashi, Keizo Tano, Akira Maruhashi, Koji Ono

**Affiliations:** a Radiation Life and Medical Science Research Division, Research Reactor Institute, Kyoto University, 2-1010, Asashiro-nishi, Kumatori-cho, Sennan-gun, Osaka, 590-0494, Japan

**Keywords:** Wortmannin, Nicotinamide, Mild temperature hyperthermia, Quiescent cell, Acute hypoxia, Chronic hypoxia

## Abstract

**Background:**

To evaluate the usefulness of fractionated administration of wortmannin combined with γ-ray irradiation in terms of local tumor response and lung metastatic potential, referring to the response of intratumor quiescent (Q) cells.

**Methods:**

B16-BL6 melanoma tumor-bearing C57BL/6 mice were continuously given 5-bromo-2’-deoxyuridine (BrdU) to label all proliferating (P) cells. The tumor-bearing mice then received γ-ray irradiation after wortmannin treatment through a single or 4 consecutive daily intraperitoneal administrations up to a total dose of 4 mg/kg in combination with an acute hypoxia-releasing agent (nicotinamide) or mild temperature hyperthermia (MTH). Immediately after the irradiation, cells from some tumors were isolated and incubated with a cytokinesis blocker. The responses of the Q and total (= P + Q) cell populations were assessed based on the frequency of micronuclei using immunofluorescence staining for BrdU. In other tumor-bearing mice, 17 days after irradiation, macroscopic lung metastases were enumerated.

**Results:**

Wortmannin raised the sensitivity of Q cells more remarkably than the total cell population in both single and daily administrations. Daily administration of wortmannin elevated the sensitivity of both the total and Q cell populations, but especially the total cell population, compared with single administration. Daily administration, especially combined with MTH, decreased the number of lung metastases.

**Conclusion:**

Daily fractionated administration of wortmannin in combination with γ-ray irradiation was thought to be more promising than single administration because of its potential to enhance local tumor response and repress lung metastatic potential.

## Introduction

It was believed that antiangiogenic therapy prevents tumor vascular growth and proliferation, thus depriving the tumor of the oxygen and nutrients necessary for survival [1]. Subsequent study, however, suggested that antiangiogenic therapy may also “normalize” the tumor vasculature for a short period of time, thereby providing a window of opportunity for improved drug delivery and enhanced sensitivity to radiation [1, 2]. The originally used approach relies on using agents that directly target vascular endothelial growth factor (VEGF) or VEGF receptor on endothelial cells. Another strategy is to indirectly target VEGF by inhibiting oncogenic signaling in cancer cells, hence decreasing both oncogenic activity and VEGF secretion [3, 4]. Inhibition of tyrosine kinase receptor signaling through RAS, phosphoinositide 3-kinase (PI-3K), and AKT was shown to result in enhanced vascular function, and this normalization enhanced tumor oxygenation and the delivery of cytotoxic drugs that might promote antitumor activity.

Tumor hypoxia results from either limited oxygen diffusion (chronic hypoxia) or limited perfusion (acute hypoxia) [5]. Further, it was reported that acute and cyclic, but not chronic, hypoxia significantly increased the number of spontaneous lung metastases, and that this effect was due to the influence of acute hypoxia treatment on the primary tumor [6, 7].

Here, using a readily metastasizing murine melanoma cell line, we tried to analyze the usefulness of combined treatment with wortmannin, originally described as a potent inhibitor of phosphoinositide 3-kinases (PI-3Ks), in radiotherapy with γ-rays in combination with an acute hypoxia-releasing agent nicotinamide or mild temperature hyperthermia (MTH), already shown to have the potential to release tumor cells from diffusion-limited chronic hypoxia [8, 9], in terms of local tumor response and lung metastatic potential. Concerning the local tumor response, the effect not only on the total (= proliferating (P) + Q) tumor cell population but also on the Q cell population was evaluated using our original method for selectively detecting the response of Q cells in solid tumors [10].

## Methods

### Mice and tumors

B16-BL6 murine melanoma cells (Institute of Development, Aging and Cancer, Tohoku University) derived from C57BL/6 mice were maintained in vitro in RPMI-1640 medium supplemented with 10% fetal bovine serum. Tumor cells (1.25 × 10^5^) were inoculated subcutaneously into the left hind leg of 8-week-old syngeneic female C57BL/6 mice (Japan Animal Co., Ltd., Osaka, Japan). Eighteen days later, the tumors, approximately 7 mm in diameter, were employed for γ-ray irradiation in this study, and the body weight of the tumor-bearing mice was 20.1 ± 2.3 (mean ± standard error) g. Mice were handled according to the Recommendations for Handling of Laboratory Animals for Biomedical Research, compiled by the Committee on Safety Handling Regulations for Laboratory Animal Experiments at our university. The p53 of B16-BL6 tumor cells is the wild type [11].

### Labeling with 5-bromo-2’-deoxyuridine (BrdU)

Twelve days after the inoculation, mini-osmotic pumps (Durect Corporation, Cupertino, CA) containing 5-bromo-2’-deoxyuridine (BrdU) dissolved in physiological saline (250 mg/mL) were implanted subcutaneously into the animals’ backs for 6 days to label all P cells. The percentage of labeled cells after continuous labeling with BrdU was 54.3 ± 6.1%, and reached a plateau at this stage. Therefore, tumor cells not incorporating BrdU after continuous labeling were regarded as Q cells.

### Treatment

After the labeling with BrdU, tumor-bearing mice received γ-ray irradiation. γ-Ray irradiation was performed with a cobalt-60 γ-ray irradiator at a dose rate of 2.5 Gy/min with tumor-bearing mice held in a specially constructed device with the tail firmly fixed with an adhesive tape. Lead blocks were used to avoid irradiating body parts other than the tumor-bearing left hind leg.

Wortmannin (C_23_H_24_O_8_, M.W. = 428.43186) dissolved in physiological saline was intraperitoneally administered to tumor-bearing mice 1 day before irradiation at a dose of 4 mg/kg, or 4 consecutive days before irradiation at a dose of 1 mg/kg. Some tumor-bearing mice further received an intraperitoneal administration of nicotinamide (1,000 mg/kg) dissolved in physiological saline 1 hour before the irradiation. Others were subjected to local mild temperature hyperthermia (MTH) at 40 °C for 60 min by immersing the implanted tumor in a water bath immediately before being irradiated [9]. Temperatures at the tumor’s center equilibrated within 3 to 4 min after immersion in the water bath and remained 0.2 - 0.3 °C below the bath’s temperature. The water bath’s temperature was maintained at 0.3 °C above the desired tumor temperature [12].

Each irradiation group also included mice that were not pretreated with BrdU.

### Immunofluorescence staining of BrdU-labeled cells and micronucleus (MN) assay

Immediately after irradiation, some tumors excised from the mice given BrdU were minced and trypsinized (0.05% trypsin and 0.02% ethylenediamine-tetraacetic acid (EDTA) in phosphate-buffered saline (PBS), 37 °C, 15 min). Tumor cell suspensions were incubated for 72 hours in tissue culture dishes containing complete medium and 1.0 µg/mL of cytochalasin-B to inhibit cytokinesis while allowing nuclear division. The cultures were trypsinized, and cell suspensions were fixed and resuspended with cold Carnoy’s fixative (ethanol:acetic acid = 3:1 in volume). Each suspension was placed on a glass microscope slide, dried at room temperature and treated with 2 M hydrochloric acid for 60 min at room temperature to dissociate the histones and partially denature the DNA. The slides were immersed in borax-borate buffer (pH 8.5) to neutralize the acid. BrdU-labeled tumor cells were detected by indirect immunofluorescence staining using a monoclonal anti-BrdU antibody (Becton Dickinson, San Jose, CA) and a fluorescein isothiocyanate (FITC)-conjugated antimouse IgG antibody (Sigma, St. Louis, MO). To distinguish the tumor cells stained with green-emitting FITC and observe them separately, cells on the slides were treated with red-emitting propidium iodide (PI, 2 µg/mL in PBS) as a background staining and monitored under a fluorescence microscope.

When cell division is disrupted, or the chromosomes are broken or damaged by chemicals or radiation, then the distribution of genetic material between the two daughter nuclei during cell division is affected and pieces or entire chromosomes fail to be included in either of the two daughter nuclei. The genetic material that is not incorporated into a new nucleus forms a “micronucleus”. Thus, the frequency of MN formation reflects the genotoxicity of a chemical compound and radiation very well. The MN frequency in cells not labeled with BrdU could be examined by counting the micronuclei in the binuclear cells that showed only red fluorescence. The MN frequency was defined as the ratio of the number of micronuclei in the binuclear cells to the total number of binuclear cells observed [10].

The ratios obtained in tumors not pretreated with BrdU indicated the MN frequency at all phases in the total tumor cell population. More than 300 binuclear cells were counted to determine the MN frequency.

### Clonogenic cell survival assay

The clonogenic cell survival assay was also performed for the implanted tumors in mice given no BrdU using an in vivo-in vitro assay method immediately after irradiation. The BrdU-unlabeled tumors were excised, weighed, minced, and disaggregated by stirring for 20 min at 37 °C in PBS containing 0.05 % trypsin and 0.02% EDTA. The cell yield was 1.2 ± 0.4 × 10^7^/g tumor weight. Appropriate numbers of viable tumor cells from the single cell suspension were plated on 60 or 100-mm tissue culture dishes, and, 12 days later, colonies were fixed with ethanol, stained with Giemsa, and counted. For the tumors that received no irradiation, the plating efficiencies for the total tumor cell populations and the MN frequencies for the total and Q cell populations are shown in Table 1. The plating efficiency indicates the percentage of cells seeded that grew into colonies when the tumors received no irradiation. The fraction of cells surviving a given dose is determined by counting the number of macroscopic colonies as a fraction of the number of cells seeded, followed by allowance, that is, dividing by the plating efficiency.

**Table 1:** Surviving Fraction and Micronucleus Frequency at 0 Gy

	Without wortmannin	With wortmannin (once)	With wortmannin (4 times)
Surviving fraction at 0 Gy (%)			
Without nicotinamide or mild temperature hyperthermia	84.4 ± 8.2^a^	69.8 ± 6.3	60.0 ± 5.5
With nicotinamide	81.4 ± 7.3	60.0 ± 5.7	55.0 ± 4.3
With mild temperature hyperthermia	83.5 ± 8.7	60.5 ± 5.3	56.0 ± 5.9
Micronucleus frequency at 0 Gy			
Total tumor cell population			
Without nicotinamide or mild temperature hyperthermia	0.050 ± 0.005	0.070 ± 0.007	0.081 ± 0.008
With nicotinamide	0.057 ± 0.006	0.082 ± 0.008	0.089 ± 0.008
With mild temperature hyperthermia	0.054 ± 0.005	0.080 ± 0.008	0.085 ± 0.009
Quiescent cells			
Without nicotinamide or mild temperature hyperthermia	0.077 ± 0.007	0.094 ± 0.009	0.102 ± 0.01
With nicotinamide	0.084 ± 0.008	0.101 ± 0.01	0.11 ± 0.01
With mild temperature hyperthermia	0.081 ± 0.008	0.099 ± 0.009	0.103 ± 0.01

^a^: Mean ± standard error (n = 9). Q cells showed significantly higher micronucleus frequencies than the total cell population under each set of conditions (P < 0.05). Wortmannin administration resulted in significantly lower surviving fractions and significantly higher micronucleus frequencies in both the total and Q cell populations than absolutely no treatment (P < 0.05).

As stated above, the MN frequencies for Q cells were obtained from BrdU-unlabeled cells in tumors after continuous BrdU labeling in vivo. The MN frequencies and surviving fractions (SFs) for total tumor cell populations were obtained from cells in tumors not pretreated with BrdU. Thus, we could not detect any interaction between BrdU and irradiation in our data for the MN frequency and SF.

### Metastasis assessment

Seventeen days after irradiation (= 35 days after the inoculation of B16-BL6 melanoma cells), the tumor-bearing mice were sacrificed by cervical dislocation, and their lungs were removed, briefly washed with distilled water, cleaned of extraneous tissue, fixed in Bouin’s solution overnight (Sigma), and stored in buffered formalin 10 % (Sigma) until metastases were counted. Macroscopically visible metastases were counted under a dissection microscope [13]. Eighteen days after the inoculation and immediately before exposure to the γ-rays, macroscopic lung metastases were also counted as background data. The number was 7.5 ± 2.2.

### Data analysis and statistics

Three mice with a tumor in the left hind leg were used to assess each set of conditions and each experiment was repeated three times. Namely, nine mice were used for each set of conditions. To examine the differences between pairs of values, Student’s t-test was used when variances of the two groups could be assumed to be equal; otherwise the Welch t-test was used. P-values are from two-sided tests. The data on cell survival and MN frequencies were fitted to the linear quadratic dose relationship [14].

## Results

Table 1 shows the surviving fractions (SFs) without γ-ray radiation for the total tumor cell population and the MN frequencies without γ-ray radiation for the total and Q cell populations. Q cells showed significantly higher MN frequencies at 0 Gy than the total cell population under each set of conditions (P < 0.05). Wortmannin administration produced significantly lower SFs and significantly higher MN frequencies at 0 Gy in both the total and Q cell populations than did absolutely no treatment (P < 0.05). The values at 0 Gy for both methods of administering wortmannin were almost the same although slightly lower SFs and higher MN frequencies were observed for the daily fractionated administration than single administration. Furthermore, the combined treatment with nicotinamide or MTH resulted in slightly lower SFs and significantly higher MN frequencies at 0 Gy in both the total and Q cell populations. The values at 0 Gy for the combined treatment with nicotinamide or MTH were almost the same although slightly lower SFs and higher MN frequencies were observed for nicotinamide than MTH.

**Figure 1:**
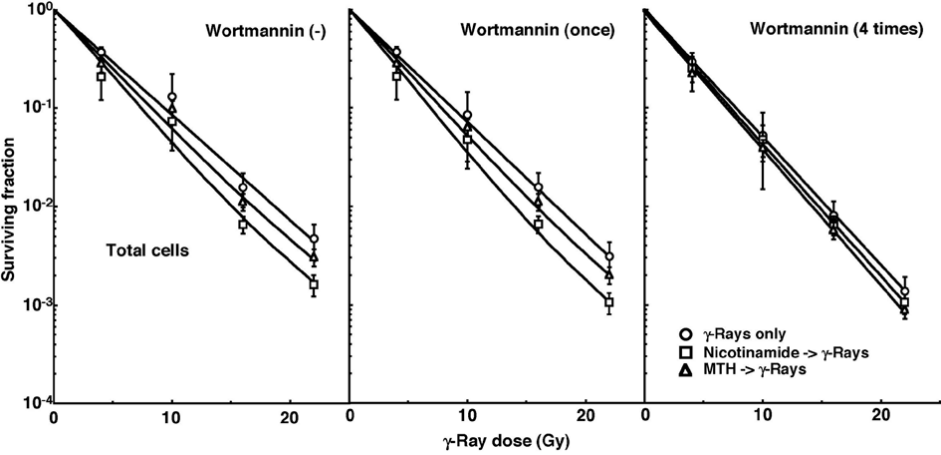
Cell survival curves for the total cell population from B16-BL6 tumors irradiated with γ-rays following single or four intraperitoneal administrations of wortmannin in combination with nicotinamide treatment or mild temperature hyperthermia (MTH) on day 18 after tumor cell inoculation. Circles, γ-ray irradiation only; Squares, γ-ray irradiation with nicotinamide treatment; Triangles, γ-ray irradiation with MTH. Bars represent standard errors (n = 9).

Cell survival curves for the total tumor cell population as a function of radiation dose are shown in Figure 1. The SFs without wortmannin and with singly administered wortmannin decreased in the following order: after γ-ray irradiation alone > after γ-ray irradiation following MTH > after γ-ray irradiation following nicotinamide administartion. Meanwhile, the SFs with daily fractionated wortmannin decreased in the order, after γ-ray irradiation alone > after γ-ray irradiation following nicotinamide administartion > after γ-ray irradiation following MTH.

**Figure 2:**
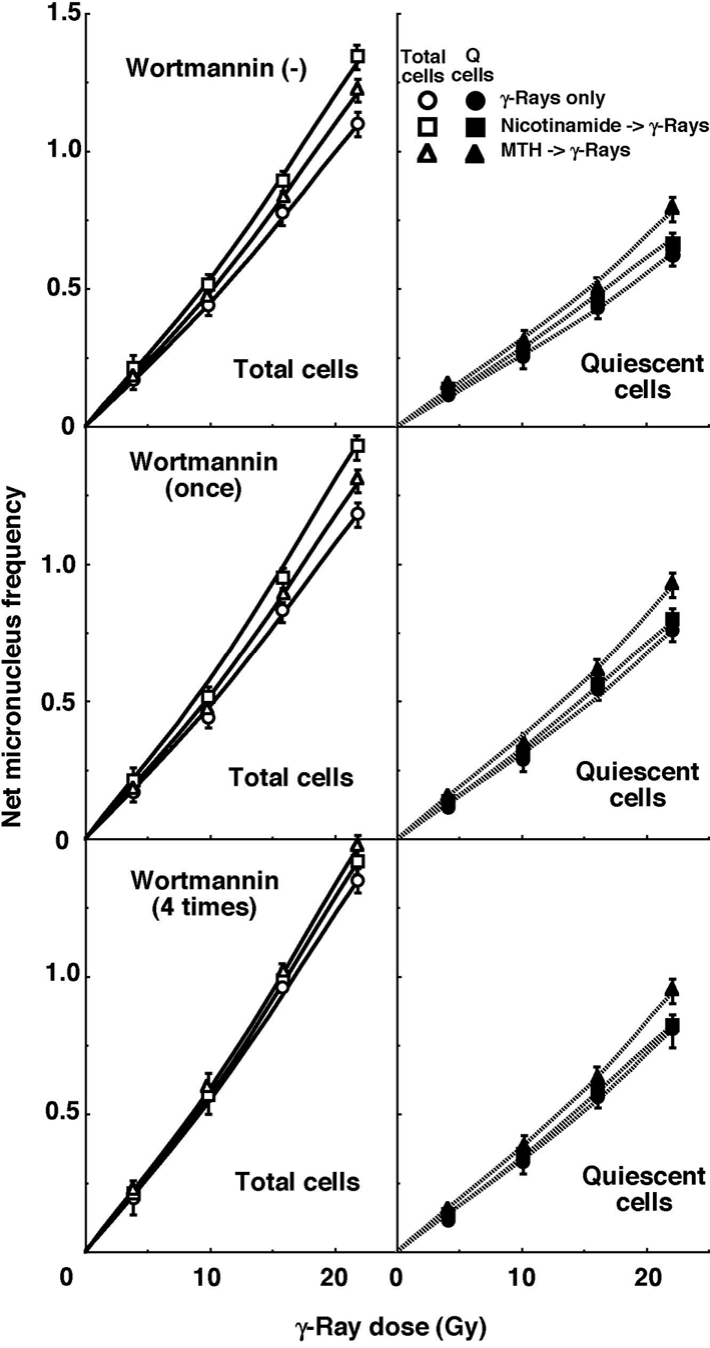
Dose response curves of the net micronucleus frequency for total (open symbols, left panels) and quiescent (solid symbols, right panels) cell populations from B16-BL6 tumors irradiated with γ-rays following single or four intraperitoneal administrations of wortmannin in combination with nicotinamide treatment or mild temperature hyperthermia (MTH) on day 18 after tumor cell inoculation. Circles, γ-ray irradiation only; Squares, γ-ray irradiation with nicotinamide treatment; Triangles, γ-ray irradiation with MTH. Bars represent standard errors (n = 9).

For baseline correction, we used the net MN frequency to exclude the MN frequency in non-irradiated tumors. The net MN frequency was defined as the MN frequency in the irradiated tumors minus that in the non-irradiated tumors. Dose response curves for the net MN frequency in total and Q tumor cell populations as a function of radiation dose are shown in Figure 2. Overall, the net MN frequencies were significantly lower in the Q cells than the total cell population (P < 0.05). In the total cell population treated without wortmannin or with singly administered wortmannin, the net MN frequency increased in the following order: after γ-ray irradiation alone < after γ-ray irradiation following MTH < after γ-ray irradiation following nicotinamide administration. In the total cell population with daily administered wortmannin and Q cell populations with or without wortmannin administration, the net MN frequency increased in the order, after γ-ray irradiation alone < after γ-ray irradiation following nicotinamide administration < after γ-ray irradiation following MTH.

To estimate the radio-enhancing effect of wortmannin, irradiation with wortmannin in both the total and Q cell populations was compared with irradiation only, using the data obtained without MTH or nicotinamide shown in Figure 1 and 2 (Table 2). The radio-enhancing effect of wortmannin was significantly larger than 1.0, and wortmannin enhanced the sensitivity of the Q cell population more than that of the total cell population, especially for single administration, with significant differences. Further, daily fractionated administration increased the sensitivity of both cell populations more than single administration, especially for the total cell population, with significant differences.

**Table 2:** Enhancement Ratios^a^ due to Combined Treatment With Wortmannin

	With wortmannin (once)	With wortmannin (4 times)
Surviving fraction = 0.03		
Total cell population	1.05 ± 0.05^b, c^	1.2 ± 0.1^c^
Net micronucleus frequency = 0.6		
Total cell population	1.05 ± 0.05^d, e^	1.2 ± 0.1^d^
Quiescent cells	1.2 ± 0.1^e^	1.3 ± 0.1

^a^: The ratio of the dose of radiation necessary to obtain each end-point without wortmannin to that needed to obtain each end-point with wortmannin; ^b^: Mean ± standard error (n = 9); Letters^c-e^ represent significant differences between two values (P < 0.05).

To estimate the radio-enhancing effect of combined treatment with nicotinamide administration or MTH in both the total and Q cell populations, the data shown in Figure 1 and 2 were used (Table 3). Without wortmannin and with singly administered wortmannin, the combination with nicotinamide and MTH had more of an enhancing effect on the total and Q cell populations, respectively, without any significant differences. However, with daily fractionated administration of wortmannin, the enhancing effect of nicotinamide was reduced, resulting in a greater effect with MTH than nicotinamide on both the total and Q cell populations.

**Table 3:** Enhancement Ratios^a^ due to Combined Treatment With Nicotinamide, or Mild Temperature Hyperthermia

	Nicotinamide	Mild temperature hyperthermia
Surviving fraction = 0.03		
Total cell population		
Without wortmannin	1.25 ± 0.1^b^	1.1 ± 0.05
With wortmannin (once)	1.2 ± 0.1	1.1 ± 0.05
With wortmannin (4 times)	1.05 ± 0.05	1.1 ± 0.05
Net micronucleus frequency = 0.6		
Total cell population		
Without wortmannin	1.2 ± 0.1	1.1 ± 0.05
With wortmannin (once)	1.2 ± 0.1	1.05 ± 0.05
With wortmannin (4 times)	1.05 ± 0.05	1.1 ± 0.05
Quiescent cells		
Without wortmannin	1.1 ± 0.05	1.2 ± 0.1
With wortmannin (once)	1.05 ± 0.05	1.15 ± 0.05
With wortmannin (4 times)	1.05 ± 0.05	1.15 ± 0.05

^a^: The ratio of the dose of radiation necessary to obtain each end-point without nicotinamide or mild temperature hyperthermia to that needed to obtain each end-point with nicotinamide or mild temperature hyperthermia; ^b^: Mean ± standard error (n = 9).

To examine the difference in radio-sensitivity between the total and Q cell populations, dose-modifying factors were calculated using the data in Figure 1 and 2 (Table 4). All the values were significantly larger than 1.0. With wortmannin administration, the difference was slightly decreased, especially with singly administered wortmannin, without any significant differences. On further combined treatment with nicotinamide and MTH, the difference in radio-sensitivity seemed to be greater and smaller, respectively, although not significantly.

**Table 4:** Dose-Modifying Factors for Quiescent Cells Relative to the Total Tumor Cell Population^a^

	Without wortmannin	With wortmannin (once)	With wortmannin (4 times)
Net micronucleus frequency = 0.6			
γ-Rays only	1.7 ± 0.15^b^	1.5 ± 0.1	1.6 ± 0.15
With nicotinamide	1.8 ± 0.15	1.65 ± 0.15	1.7 ± 0.15
With mild temperature hyperthermia	1.6 ± 0.15	1.4 ± 0.1	1.55 ± 0.1

^a^: The ratio of the dose of radiation necessary to obtain each end-point in the quiescent cell population to that needed to obtain each end-point in the total tumor cell population; ^b^: Mean ± standard error (n = 9).

**Figure 3:**
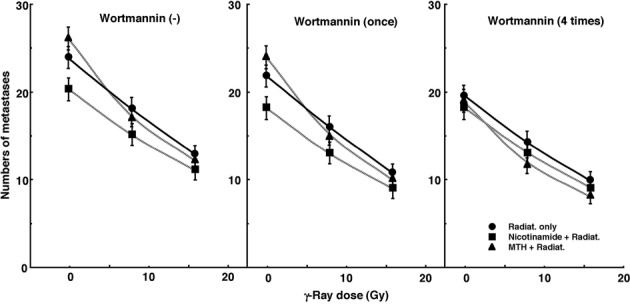
Cell survival curves for the total cell population from B16-BL6 tumors irradiated with γ-rays following single or four intraperitoneal administrations of wortmannin in combination with nicotinamide treatment or mild temperature hyperthermia (MTH) on day 18 after tumor cell inoculation. Circles, γ-ray irradiation only; Squares, γ-ray irradiation with nicotinamide treatment; Triangles, γ-ray irradiation with MTH. Bars represent standard errors (n = 9).

Figure 3 shows the numbers of lung metastases on day 35 after inoculation as a function of the absorbed dose of γ-ray irradiation with or without wortmannin administration in combination with nicotinamide administration or MTH. Without irradiation, the nicotinamide combination seemed to decrease the numbers of macroscopic metastases. With irradiation, as the absorbed dose increased, the numbers decreased. Without wortmannin and with singly administered wortmannin, the numbers decreased more remarkably with nicotinamide. However, with daily fractionated administration of wortmannin, the numbers decreased more remarkably with MTH. Namely, for no administration of wortmannin and the single administration of wortmannin, the combination with nicotinamide, and for the daily administration of wortmannin, the combination with MTH, which were most cytotoxic as an initial effect in a clonogenic cell survival assay, seemed to reduce the numbers of lung metastases from the local tumors most efficiently.

The numbers of lung metastases from local tumors that received irradiation under each set of conditions, which produced an identical SF of 0.03 as an initial effect (Figure 1), were estimated using the data shown in Figure 3 (Table 5). Overall, the combination with wortmannin tended to decrease the numbers more than γ-ray irradiation only, especially with daily fractionated administration. The combination of nicotinamide without wortmannin or with singly administered wortmannin resulted in a slightly smaller number than without nicotinamide, although the difference was not significant. Further, the combination of MTH with daily administration of wortmannin led to a slightly smaller number than without MTH, though again not significantly. Thus, when wortmannin was administered daily combined with MTH, the number was significantly smaller than that for γ-ray irradiation only.

**Table 5:** The Numbers of Metastases From the Irradiated Tumors That Received Cytotoxic Treatment Producing a Similar Initial Local Effect^a^

	Without wortmannin	With wortmannin (once)	With wortmannin (4 times)
Surviving fraction = 0.03			
γ-Rays only	14.2 ± 1.4^b, c, d, e^	12.4 ± 1.3	12.0 ± 1.2
With nicotinamide	13.3 ± 1.3	11.5 ± 1.2^c, f^	11.4 ± 1.2^d, g^
With mild temperature hyperthermia	14.2 ± 1.4^f, g, h^	12.2 ± 1.2	10.7 ± 1.1^e, h^

^a^: Based on the data shown in Figure 3, the estimated numbers of lung metastatic nodules from local tumors that received γ-ray irradiation with or without wortmannin in combination with nicotinamide or mild temperature hyperthermia, which produced an identical surviving fraction of 0.03 as an initial effect on Figure 1;^b^: Mean ± standard error (n = 9); Letters^c-h^ represent significant differences between two values (P < 0.05).

## Discussion

Two major pathways for the repair of potentially lethal DNA double-stranded breaks (dsbs) exist in mammalian cells. The non-homologous end-joining (NHEJ) pathway is imprecise, error-prone, and mutagenic, and mutant cell lines lacking key components of this pathway all exhibit impaired kinetics of DNA dsb repair and exquisite radiosensitivity [15, 16]. Homologous recombination (HR) is a more precise (error-free) repair mechanism and is more important for the repair of dsbs in late-S and G2 when a sister chromatid is available for the recombination reaction. Cell lines with defects in HR also exhibit increased radiosensitivity and decreased fidelity of repair [15, 16].

Wortmannin was originally described as a potent inhibitor of phosphoinositide 3-kinases (PI-3Ks), which are lipid kinases involved in insulin and other mitogenic signaling pathways. The kinase domain of PI-3K shares homology with ATM protein, which is a protein kinase that functions in DNA damage-responsive signaling pathways by phosphorylating target proteins [17]. ATM is related to several other protein kinases involved in the regulation of eukaryotic cell cycle progression and DNA damage-triggered responses [17, 18]. These proteins all contain a carboxy-terminal kinase domain that shares significant sequence homology with the kinase domains of mammalian and yeast PI-3Ks. In this family of PI-3K-related kinases, there is a catalytic subunit of the DNA-dependent protein kinase (DNA-PKcs) that functions in the NHEJ DNA repair pathway. Thus, wortmannin is thought to show the potential to inhibit the catalytic activities of DNA-PKcs, thereby hampering the NHEJ repair pathway which functions all through the cell cycle irrespective of the proliferation status of cells [18]. Thus, wortmannin showed more of an enhancing effect on non-proliferating Q cells than on the total cell population composed of Q and P cells including cells in the late-S and G2 phases where the HR repair pathway is dominant (Table 2). However, wortmannin is also known to inhibit the PI3K-Akt-mTOR signaling pathway which facilitates the proliferation and survival of cells [17]. Thus, in order to analyze the involvement of the PI3K signaling pathway, DNA-PK and/or ATM knockdown studies are needed in the future.

Tumor hypoxia can be manipulated by the treatment with an acute hypoxia-releasing agent, nicotinamide through its inhibiting action on temporary fluctuations in tumor blood flow [8, 9] or MTH, already shown to have the potential to release tumor cells from diffusion-limited chronic hypoxia [8, 9]. Taking into account our previous finding that the total and Q cell populations of B16-BL6 tumors are predominantly composed of acute and chronic hypoxia, respectively [9], it seems reasonable that the combination with nicotinamide and MTH had a greater enhancing effect on the total and Q cell populations, respectively, in the tumors treated without wortmannin or with singly administered wortmannin (Table 3). However, in the tumors treated daily with wortmannin, the effect of nicotinamide was reduced, leading to a greater enhancing effect of MTH than nicotinamide on both the total and Q cell populations (Table 3). Thus, it follows that the daily administration of wortmannin had already released cells from acute hypoxia before the nicotinamide treatment.

In our previous report, it was shown that the anti-VEGF agent bevacizumab has the potential to reduce perfusion-limited acute hypoxia during a vascular normalization window lasting some 2 to 5 days after the blocking of VEGF [19]. Irrespective of the same total dose of administered wortmannin, the single administration 1 day before irradiation could not release acute hypoxia, but daily fractionated administration before irradiation could. Therefore, it can be thought that the indirect targeting of VEGF by inhibiting oncogenic signaling through PI-3K decreased VEGF secretion, resulting in enhanced vascular function, and that this normalization enhanced tumor oxygenation including reducing acute hypoxia. During this normalization window induced by inhibiting oncogenic signaling through PI-3K, improvements in tumor oxygenation might promote antitumor activity [3, 4, 20]. Actually, even without the combined treatment with nicotinamide or MTH, the enhancing effect of wortmannin on both the total and Q cell populations was increased through daily fractionated administration (Table 2). In future, we would like to analyze this normalization window further.

The presence of Q cells is probably due, at least in part, to hypoxia and the depletion of nutrition, a consequence of poor vascular supply [21, 22]. As a result, Q cells are viable and clonogenic, but have ceased dividing. This might promote the formation of micronuclei at 0 Gy in Q tumor cells (Table 1). Q cells were shown to have significantly less radiosensitivity than the total cell population [10, 21, 22], that is, more Q cells survive radiotherapy than P cells (Fig. 2, Table 4). Thus, the control of chronic hypoxia-rich Q cells has a great impact on the outcome of conventional radiotherapy for controlling local tumors, resulting in the superiority of the combination of wortmannin and MTH in radiotherapy. As a result, the combined use of wortmannin and MTH led to a decrease in the difference in radiosensitivity (Table 4). In contrast, the combined use of wortmannin and nicotinamide which has an enhancing effect on the acute hypoxia-rich total cell population led to an increase in the difference in radiosensitivity. Further, when wortmannin was administered in a daily fractionated manner which also has the potential to release cells from acute hypoxia, the difference in radiosensitivity was slightly increased.

Hypoxia is suggested to enhance metastasis by increasing genetic instability [6, 7]. Acute but not chronic hypoxia increased the number of macroscopic metastases in mouse lungs [6, 7]. We recently reported the significance of the administration of an acute hypoxia-releasing agent, nicotinamide, into tumor-bearing mice as a combined treatment with γ-ray irradiation in terms of repressing lung metastasis [9]. With or without irradiation, nicotinamide and the VEGF blocking treatment reduced the number of macroscopic lung metastases (Fig. 3, Table 5). During the window of vascular normalization, acute hypoxia may be released and this release is more important in suppressing metastasis from the primary tumor than is the release of cells from chronic hypoxia. Without irradiation, MTH slightly increased the number of metastases, implying that the release from chronic hypoxia is not as important in repressing metastasis as the release from acute hypoxia. However, hyperthermia is not thought to induce metastasis in the clinical setting [23]. Meanwhile, as the dose of γ-rays increased with irradiation, the number of macroscopic lung metastases decreased reflecting the decrease in the number of clonogenically viable tumor cells in the primary tumor (Fig. 3). The metastasis-repressing effect of the release from acute hypoxia without irradiation became less distinct with irradiation. This is partly because the metastasis-repressing effect achieved through a reduction in the number of clonogenic tumor cells by irradiation is much greater than that achieved by releasing tumor cells from acute hypoxia. An acute hypoxia-releasing treatment may be promising for reducing numbers of lung metastases.

### Conclusion

It was elucidated that control of the chronic hypoxia-rich Q cell population in primary solid tumors has the potential to impact the control of local tumors as a whole, while control of the acute hypoxia-rich total tumor cell population has the potential to impact the control of lung metastases. Namely, in conventional radiotherapy, daily fractionated administration of wortmannin combined with MTH is thought to have a great potential to control both local solid tumors and lung metastases from the local tumors.

## Declaration

Authors have no conflict of interest concerning this manuscript. The authors have no conflict of interest to declare.
